# Prediction of bacteremia using routine hematological and metabolic parameters based on logistic regression and random forest models

**DOI:** 10.3389/fcimb.2025.1605485

**Published:** 2025-07-28

**Authors:** Ting-Qiang Wang, Ying Zhuo, Chun-E Lv, Jing Shi, Ling-Hui Yao, Shi-Yan Zhang, Jinbao Shi

**Affiliations:** ^1^ Department of Clinical Laboratory, Fuding Hospital, Fujian University of Traditional Chinese Medicine, Fuding, Fujian, China; ^2^ Department of Anesthesiology, Fuding Hospital, Fujian University of Traditional Chinese Medicine, Fuding, Fujian, China; ^3^ Department of Nephrology, Ningde Hospital of Traditional Chinese Medicine, Ningde, Fujian, China

**Keywords:** bacteremia, blood culture, machine learning, random forest, logistic regression, biomarkers

## Abstract

**Background:**

This study aimed to evaluate the predictive utility of routine hematological, inflammatory, and metabolic markers for bacteremia and to compare the classification performance of logistic regression and random forest models.

**Methods:**

A retrospective study was conducted on 287 inpatients who underwent blood culture testing at Fuding Hospital, Fujian University of Traditional Chinese Medicine between March and August 2024. Patients were divided into bacteremia (n = 137) and non-bacteremia (n = 150) groups based on blood culture results. Hematological indices, inflammatory markers (e.g., C-reactive protein (CRP), procalcitonin (PCT)), metabolic indices (e.g., glucose, cholesterol) and nutritional markers (e.g., albumin) were analyzed. Univariate and multivariate binary logistic regression analyses were used to identify independent risk factors. Logistic regression and random forest models were developed using 33 features with a 70:30 train-test split and evaluated using the receiver operating characteristic (ROC) curves, confusion matrices and standard classification.

**Results:**

Hemoglobin, cholesterol, and albumin levels were significantly lower in the bacteremia group, while platelet count, CRP, PCT, glucose, and triglycerides were significantly elevated (all p < 0.05). Logistic regression identified platelet count (Odds ratios (OR) = 1.003, 95% confidence interval (CI): 1.001–1.006), PCT (OR = 1.032, 95% CI: 1.004–1.060), triglycerides (OR = 1.740, 95% CI: 1.052–2.879), and low cholesterol (OR = 0.523, 95% CI: 0.383–0.714) as independent risk factors. The area under the ROC curve (AUC) was 0.75 for the random forest model and 0.74 for logistic regression, with recall rates of 0.69 and 0.60, respectively.

**Conclusion:**

Routine laboratory markers integrated into machine learning models demonstrated potential for early bacteremia prediction. Random forest exhibited superior sensitivity compared to logistic regression, suggesting its potential utility as a clinical screening tool.

## Background

Bacteremia is a systemic infection resulting from the invasion of pathogenic microorganisms into the bloodstream. Without prompt recognition and treatment, it can progress rapidly to sepsis or septic shock, with mortality rates ranging between 30% and 50% (Rudd et al., 2020). While blood culture remains the diagnostic gold standard, it is hindered by delayed turnaround times (24–72 hours) and reduced sensitivity, especially when prior antibiotic exposure or contamination occurs. These limitations can delay critical treatment decisions (Evans et al., 2021). As a result, there is growing demand for rapid, cost-effective tools to support early bacteremia detection and guide clinical management.

In recent years, routine hematological parameters, such as white blood cell count and the neutrophil-to-lymphocyte ratio, and metabolic markers like glucose and cholesterol have gained attention for their utility in infection detection due to their accessibility and affordability (Zhang et al., 2024). These biomarkers offer partial insight into host inflammatory responses and infection-induced metabolic alterations, with studies demonstrating their moderate diagnostic sensitivity and specificity in infectious disease contexts (Agnello et al., 2021; Gatica et al., 2023; Zhang et al., 2024). However, individual parameters lack predictive power due to complex, nonlinear interactions in systemic infections (Moor et al., 2023). Recent studies have explored the use of machine learning approaches for early bacteremia and sepsis prediction, demonstrating improved risk stratification compared to traditional clinical scores (Moor et al., 2021; Yan et al., 2022; Chua et al., 2025). Machine learning models, which capture such interactions, remain underexplored in resource-limited settings.

This study integrated routine hematological, inflammatory, and metabolic markers to develop logistic regression and random forest prediction models. The primary objective was to evaluate the comparative performance of these models in the early identification of bacteremia and to explore a simple, practical tool for clinical screening support. Additionally, the findings may provide a theoretical foundation for the broader application of such predictive models across diverse healthcare settings.

## Materials and methods

### Study population

This pilot study retrospectively included 287 hospitalized patients who underwent blood culture testing at Fuding Hospital, Fujian University of Traditional Chinese Medicine, between March and August 2024. Based on the results of blood culture, patients were classified into two groups: the bacteremia group (culture-positive, n = 137) and the non-bacteremia group (culture-negative, n = 150). The sample size reflects all eligible patients meeting the inclusion and exclusion criteria during the specified period, representing the entire population available for analysis at the study center.

### Inclusion and exclusion criteria

#### Inclusion criteria

Patients who underwent at least one blood culture during hospitalization; Availability of complete clinical and laboratory data.

#### Exclusion criteria

1. Suspected contamination in blood culture results (e.g., common skin flora);
2. Antibiotic use within the preceding three days;
3. Coexisting severe hematological disorders or immunodeficiency;
4. Missing key clinical or laboratory parameters;
5. Blood culture-positive cases lacking clinical signs of infection (e.g., absence of fever, hypotension, or organ dysfunction).

#### Diagnostic criteria

Diagnosis of bacteremia followed the Third International Consensus Definitions for Sepsis and Septic Shock (Sepsis-3) (Seymour et al., 2016), and was established if one or more of the following criteria were met: (1) body temperature > 38 °C or <36 °C; (2) systolic blood pressure < 90 mmHg or a drop > 40 mmHg from baseline; (3) isolation of pathogenic organisms from blood cultures (in the case of skin commensals, at least two positive cultures from separate draws were required); (4) evidence of organ dysfunction, such as a Sequential Organ Failure Assessment (SOFA) score ≥ 2.

### Ethical approval and compliance

The study protocol was approved by the Ethics Committee of Fuding Hospital, Fujian University of Traditional Chinese Medicine (Approval No. 2023012). All data were anonymized prior to analysis to ensure compliance with ethical and privacy standards. Due to the retrospective nature of the study, the requirement for written informed consent was waived by the ethics committee.

### Data collection and laboratory Indicators

Clinical and laboratory data were extracted from the hospital information system and laboratory information system. The following indicators were collected:

Hematological parameters: red blood cell count, hemoglobin, white blood cell count, neutrophils, lymphocytes, platelet count, mean platelet volume (MPV), platelet distribution width (PDW).

Inflammatory markers: CRP and PCT.

Metabolic markers: fasting blood glucose, total cholesterol, triglycerides, uric acid.

Nutritional marker: albumin.

Categorical variables: sex, smoking history, drinking history, hypertension, coronary heart disease, tumor history, diabetes, site of infection.

### Laboratory procedures and equipment

All blood samples were obtained prior to the initiation of antimicrobial therapy. Hematological analyses were performed using the Sysmex XN-9000 automated hematology analyzer (Japan). Biochemical markers (glucose, cholesterol, triglycerides, uric acid, albumin) were measured with the Beckman AU5800 automated chemistry analyzer. CRP and PCT levels were assessed using electrochemiluminescence immunoassay. Blood cultures were processed using the BacT/ALERT 3D automated system and pathogen identification was performed with the VITEK MS mass spectrometry system (bioMérieux, France).

Blood cultures were collected using a bilateral dual-bottle approach (aerobic and anaerobic pairs from both arms). If common skin flora such as coagulase-negative *staphylococci* or *Propionibacterium* species were isolated, at least two positive cultures from separate sites or repeated collections were required to confirm true infection rather than contamination.

All procedures strictly followed standard operating protocols (SOPs), with internal quality control maintained and coefficients of variation kept below 5%. The present study was centered on bacterial pathogens retrieved from blood cultures. To maintain the research’s focus on bacterial infections and ensure the consistency of statistical analysis, fungi, *Mycoplasma*, *Chlamydia*, *parasites*, and viruses were excluded.

### Statistical analysis

Normality of continuous variables was assessed using the Shapiro-Wilk test. Normally distributed data were expressed as mean ± standard deviation and compared using independent samples t-test. Non-normally distributed data were expressed as median (interquartile range, P25 - P75) and analyzed using the Mann-Whitney U test. Categorical variables were presented as frequencies and percentages, with between-group comparisons performed using the chi-square (χ^2^) test.

To identify risk factors for bacteremia, univariate logistic regression was first conducted. Variables with p < 0.20 were included in the multivariate logistic regression model, and backward stepwise elimination was used to determine independent predictors. Odds ratios (ORs) with 95% confidence intervals (CIs) were reported.

### Model development and performance evaluation

Both the logistic regression and random forest models included 33 features, comprising 22 continuous variables (e.g., age, complete blood count indices, CRP, PCT, glucose, cholesterol) and 11 one-hot encoded categorical variables (e.g., sex, comorbidities, site of infection).

The dataset was randomly divided into a training set (n = 201) and a testing set (n = 86) in a 70:30 ratio, with stratification to maintain the proportion of bacteremia cases in both subsets.

Logistic regression model: L2 regularization was applied. Model tuning included optimization of the regularization parameter C (range: 0.001 - 100) and solver type (liblinear, lbfgs).

Random forest model: Hyperparameter optimization was performed via grid search across n_estimators (50, 100, 200), max_depth (None, 10, 20), min_samples_split (2, 5, 10), and min_samples_leaf (1, 2, 4), using five-fold stratified cross-validation. The final model selected 200 decision trees with a maximum tree depth of 20.

Performance metrics included accuracy, precision, recall, F1 score, and AUC.

All traditional statistical analyses were performed using SPSS version 22.0. Machine learning model development and evaluation were conducted in Python 3.7 using the Scikit-learn library. A two-tailed p-value < 0.05 was considered statistically significant.

## Results

### Shapiro–Wilk test for normality

The Shapiro-Wilk test was applied to assess the distribution of all continuous variables. Results indicated that variables such as hemoglobin, MPV, and albumin conformed to a normal distribution in both groups (p > 0.05). In contrast, most other variables, including CRP, blood glucose, and PCT, exhibited significant deviations from normality (p < 0.05). Accordingly, parametric or non-parametric statistical methods were applied as appropriate in subsequent analyses.

### Baseline characteristics of study participants

Baseline characteristics of the bacteremia and non-bacteremia groups are summarized in **Table 1**. The proportion of female patients was significantly higher in the bacteremia group compared to the non-bacteremia group (47.4% vs. 34.0%, p = 0.020). Additionally, the prevalence of diabetes mellitus was significantly elevated in the bacteremia group (32.1% vs. 20.7%, p = 0.027). Other variables, including age, hypertension, coronary artery disease, malignancy, alcohol consumption, and smoking history, showed no statistically significant differences between groups (all p > 0.05), indicating overall comparability of the two populations with respect to most baseline comorbidities.

**Table 1:** Baseline characteristics of patients in bacteremia and non-bacteremia groups [n (%)].

Variable	Category	Non-bacteremia group (N = 150)	Bacteremia group (N = 137)	χ^2^/U Statistic	P-value
Sex	Female	51 (34.00)	65 (47.40)	5.375	0.020
	Male	99 (66.00)	72 (52.60)		
Alcohol consumption	No	128 (85.30)	126 (92.00)	3.100	0.078
	Yes	22 (14.70)	11 (8.00)		
Smoking	No	132 (88.00)	129 (94.20)	3.299	0.069
	Yes	18 (12.00)	8 (5.80)		
Hypertension	No	52 (40.00)	59 (43.10)	0.005	0.946
	Yes	78 (60.00)	72 (61.00)		
Coronary artery disease	No	135 (90.00)	127 (92.70)	0.657	0.418
	Yes	15(10.00)	10 (7.30)		
Malignancy	No	136 (90.70)	123 (89.80)	0.064	0.801
	Yes	14 (9.30)	14 (10.20)		
Diabetes mellitus	No	119 (79.30)	93 (67.90)	4.863	0.027
	Yes	31 (20.70)	44 (32.10)		
Age (years)	Median (P25-P75)	68.00 (58.00 - 76.00)	66.00 (56.00 - 76.00)	- 0.843	0.399
	Range	22.00 - 92.00	2.00 - 95.00		

Group comparisons were conducted using chi-square tests for categorical variables and Mann–Whitney U tests for non-normally distributed continuous variables. P25: 25th percentile; P75: 75th percentile.

### Comparison of clinical laboratory parameters

As shown in **Table 2**, patients in the bacteremia group had significantly lower levels of hemoglobin, red blood cell count, cholesterol, and albumin compared to those in the non-bacteremia group (all p < 0.01). Conversely, levels of platelet count, CRP, PCT, blood glucose, and triglycerides were significantly higher in the bacteremia group (all p < 0.05). No statistically significant differences were observed in other parameters such as mean corpuscular hemoglobin concentration (MCHC) and platelet distribution width (PDW).

**Table 2:** Comparison of laboratory parameters between the bacteremia and Non-bacteremia groups [median (P25–P75)].

Parameter	Non-bacteremia group (N = 150)	Bacteremia group (N = 137)	Z-value	P-value
RBC (×10^12^/L)	4.08 (3.61 - 4.97)	3.72 (3.27 - 4.21)	-4.063	< 0.001
Hemoglobin (g/L)	120.00 (103.00 - 137.00)	108.00 (94.50 - 122.00)	-4.029	< 0.001
MCH (pg)	29.65 (28.10 - 30.80)	29.10 (27.95 - 30.40)	-1.216	0.224
MCV(fL)	90.30 (87.23 - 93.88)	90.00 (86.00 - 94.00)	-0.431	0.667
RDW (%)	13.20 (12.40 - 15.10)	13.40 (12.50 - 15.10)	-1.036	0.300
WBC (×10^9^/L)	6.64 (5.23 - 9.08)	7.66 (5.58 - 9.60)	-1.820	0.069
Neutrophils (×10^9^/L)	4.36 (3.00 - 6.39)	5.33 (3.31 - 7.16)	-1.917	0.055
Lymphocytes (×10^9^/L)	1.47 (1.08 - 2.00)	1.39 (0.96 - 1.83)	-1.324	0.186
Eosinophils (×10^9^/L)	0.13 (0.06 - 0.20)	0.11 (0.05 - 0.18)	-0.777	0.437
Basophils (×10^9^/L)	0.03 (0.02 - 0.05)	0.04 (0.02 - 0.05)	-0.367	0.714
Platelets (×10^9^/L)	246.50 (189.00 - 309.00)	282.00 (208.00 - 383.00)	-2.817	0.005
PDW (%)	10.60 (9.40-12.30)	10.50 (9.20 - 13.40)	-0.189	0.850
MPV (fL)	9.80 (9.20 - 10.40)	9.60 (9.00 - 10.35)	-1.161	0.246
CRP (mg/L)	14.04 (5.10 -34.64)	19.72 (6.76 - 52.40)	-2.390	0.017
Procalcitonin (ng/mL)	0.11 (0.06 - 0.34)	0.20 (0.09 - 0.77)	-3.655	< 0.001
Glucose (mmol/L)	5.40 (4.78 - 7.25)	5.88 (5.00 - 8.41)	-2.251	0.024
Cholesterol (mmol/L)	4.00 (3.34 - 4.70)	3.53 (3.07 - 4.04)	-3.663	< 0.001
Triglycerides (mmol/L)	1.19 (0.80 - 1.38)	1.31 (0.90 - 1.55)	-2.416	0.016
Uric acid (μmol/L)	270.00 (189.00-353.00)	221.00 (169.00-297.00)	-2.832	0.005
Albumin (g/L)	35.65 (32.20 - 37.90)	33.30 (30.50 - 36.50)	-3.666	<0.001

As data were non-normally distributed, group comparisons were conducted using the non-parametric Mann–Whitney U test. P25, 25th percentile; P75, 75th percentile; RBC, Red blood cells; MCH, mean corpuscular hemoglobin; MCV, mean corpuscular volume; RDW, red cell distribution width; WBC, White blood cells; PDW, platelet distribution width; MPV, mean platelet volume; CRP, C-reactive protein.

### Univariate and multivariate logistic regression analysis

As shown in **Table 3**, univariate logistic regression analysis revealed that several variables - including hemoglobin, platelet count, PCT, blood glucose, cholesterol, albumin, and diabetes mellitus - were significantly associated with bacteremia. These candidate variables were subsequently entered into a multivariate logistic regression model to identify independent predictors (**Table 4**).

**Table 3:** Univariate logistic regression analysis for predictors of bacteremia.

Variable	B (Coefficient)	SE	Wald χ^2^	P-value	OR (95% CI)
Hemoglobin (g/L)	-0.021	0.005	15.209	<0.001	0.979 (0.969 - 0.990)
MCH (pg)	-0.035	0.043	0.669	0.413	0.965 (0.887 - 1.051)
MCV (fL)	-0.003	0.016	0.035	0.851	0.997 (0.965 - 1.030)
MCHC (g/L)	-0.014	0.010	2.019	0.155	0.987 (0.968 - 1.005)
RDW (%)	0.058	0.048	1.432	0.231	1.059 (0.964 - 1.165)
WBC (×10^9^/L)	0.066	0.032	4.113	0.043	1.068 (1.002 - 1.138)
Neutrophils (×10^9^/L)	0.074	0.033	4.920	0.027	1.076 (1.009 - 1.149)
Lymphocytes (×10^9^/L)	-0.177	0.166	1.132	0.287	0.838(0.604 - 1.161)
Eosinophils (×10^9^/L)	-0.323	0.633	0.260	0.610	0.724(0.210 - 2.502)
Basophils (×10^9^/L)	-0.378	4.922	0.006	0.939	0.685 (0.000 -10608.263)
Platelets (×10^9^/L)	0.003	0.001	8.271	0.004	1.003 (1.001 - 1.005)
PDW (%)	0.042	0.049	0.763	0.382	1.043 (0.949 - 1.148)
MPV (fL)	-0.098	0.122	0.637	0.425	0.907 (0.714 - 1.153)
CRP (mg/L)	0.004	0.002	3.058	0.080	1.004 (0.999-1.009)
PCT (ng/mL)	0.020	0.014	2.187	0.139	1.021 (0.993 - 1.049)
Glucose (mmol/L)	0.124	0.042	8.680	0.003	1.132 (1.042-1.230)
Cholesterol (mmol/L)	-0.464	0.125	13.886	<0.001	0.628 (0.492-0.802)
Triglycerides (mmol/L)	0.284	0.197	2.064	0.151	1.328 (0.902 - 1.955)
Uric acid (μmol/L)	-0.002	0.001	5.376	0.020	0.998 (0.996-1.000)
Albumin (g/L)	-0.095	0.027	12.126	<0.001	0.909 (0.862-0.959)
Age (years)	-0.009	0.007	1.431	0.232	0.991 (0.977 - 1.006)
Sex	-0.561	0.243	5.336	0.021	0.571 (0.355 - 0.919)
Alcohol consumption	-0.677	0.390	3.017	0.082	0.508 (0.236 - 1.091)
Smoking	-0.788	0.443	3.169	0.075	0.455 (0.191 - 1.083)
Hypertension	0.016	0.239	0.005	0.946	1.016 (0.637 - 1.623)
Coronary artery disease	-0.344	0.427	0.652	0.419	0.709 (0.307 - 1.635)
Malignancy	0.100	3.398	0.064	0.801	1.106 (0.507 - 2.412)
Diabetes mellitus	0.597	0.272	4.803	0.028	1.816 (1.065 - 3.097)
Site of infection					
Head	-0.981	0.709	1.913	0.167	0.375 (0.093 - 1.505)
Respiratory tract	-2.175	0.557	15.271	<0.001	0.114 (0.038 - 0.338)
Gastrointestinal tract	-0.685	0.554	1.528	0.216	0.504 (0.170 - 1.494)
Urinary tract	0.288	0.719	0.160	0.689	1.333 (0.326 - 5.455)

CI, confidence interval; PDW, platelet distribution width; RDW, red cell distribution width; MCH, mean corpuscular hemoglobin; WBC, White blood cells; MCV, mean corpuscular volume; MCHC, mean corpuscular hemoglobin concentration; MPV, mean platelet volume; CRP, C-reactive protein; PCT, Procalcitonin.

**Table 4:** Multivariate logistic regression analysis for independent predictors of bacteremia.

Variable	Reference	B (Coefficient)	SE	Wald χ^2^	P -value	OR (95% CI)
MCHC (g/L)		-0.019	0.011	2.708	0.100	0.982 (0.960 - 1.004)
Platelet (×10^9^/L)		0.003	0.001	6.654	0.010	1.003 (1.001 - 1.006)
Procalcitonin (ng/mL)		0.031	0.014	5.181	0.023	1.032 (1.004 - 1.060)
Glucose (mmol/L)		0.091	0.049	3.363	0.067	1.095 (0.994 - 1.206)
Cholesterol (mmol/L)		-0.648	0.159	16.654	0.000	0.523 (0.383 - 0.714)
Triglycerides (mmol/L)		0.554	0.257	4.650	0.031	1.740 (1.052 - 2.879)
Sex	Female	-0.497	0.301	2.727	0.099	0.608(0.337 -1.097)
Site of infection	Other					
Head		-1.192	0.591	4.062	0.044	0.304 (0.095 - 0.968)
Respiratory tract		0.407	0.585	0.484	0.487	1.503 (0.477 - 4.734)
Gastrointestinal tract		1.212	0.757	2.563	0.109	3.359 (0.762 -14.811)
Urinary tract		0.528	0.764	0.478	0.489	1.695 (0.379 - 7.578)
Constant		6.550	3.822	2.937	0.087	698.993

CI, confidence interval; MCHC, mean corpuscular hemoglobin concentration.

The multivariate analysis identified the following as statistically significant independent risk factors for bacteremia: Platelet count (OR = 1.003, p = 0.010); PCT (OR = 1.032, p = 0.023); Triglycerides (OR = 1.740, p = 0.031); Cholesterol (OR = 0.523, p < 0.001).

### Performance evaluation of machine learning models

**Figure 1:**
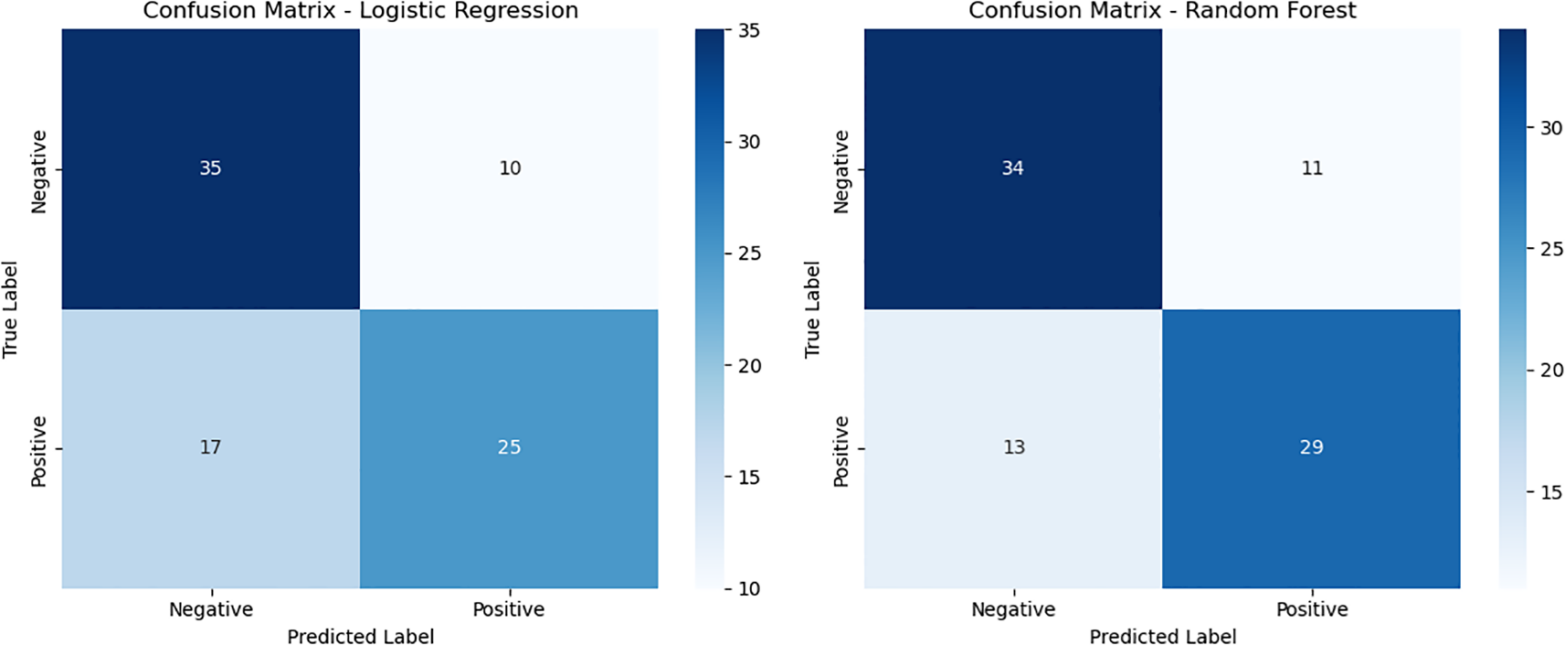
Confusion matrices of logistic regression and random forest models. This figure illustrates the confusion matrices for the logistic regression model (left) and the random forest model (right) on the test dataset. Compared with logistic regression, the random forest model achieved a slightly higher number of true positives (TP = 29 vs. 25) and fewer false negatives (FN = 13 vs. 17), indicating improved sensitivity. However, the random forest model also showed a modest increase in false positives (FP = 11 vs. 10), suggesting a slight reduction in specificity as a trade-off for higher sensitivity.

**Figure 2:**
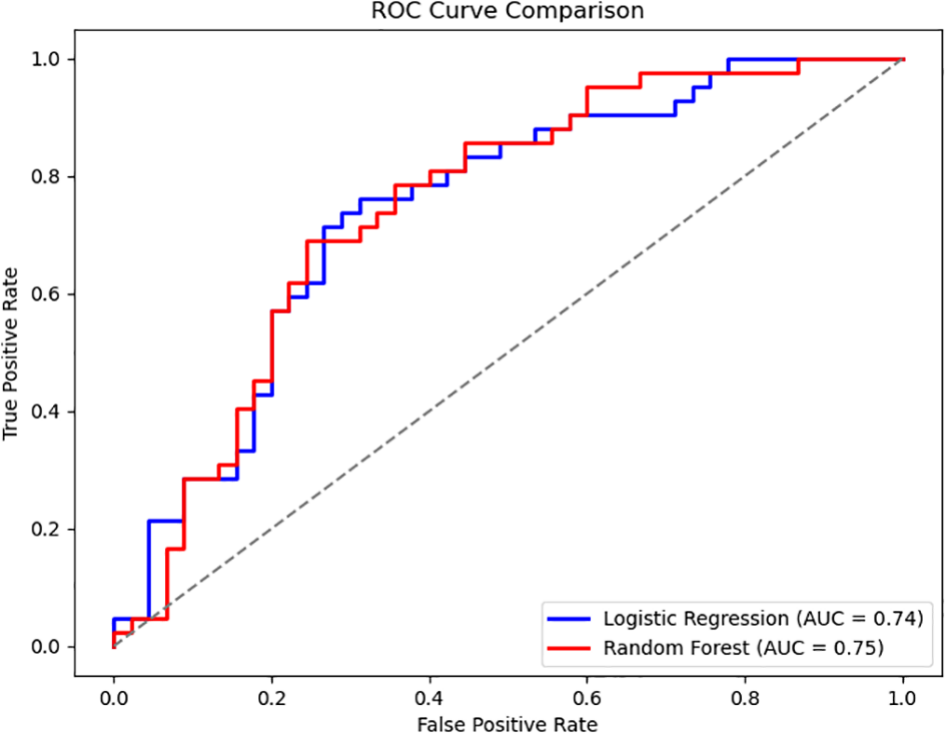
Comparison of ROC curves between logistic regression and random forest models. The ROC curves of the two models exhibit similar shapes, indicating that logistic regression and random forest achieved comparable classification performance on this dataset.

The classification performance of the logistic regression and random forest models on the testing set is presented in **Tables 5**, **6**. The logistic regression model achieved an accuracy of 0.69, with a recall rate of 0.60 for the bacteremia (positive) group and an area under the ROC curve (AUC) of 0.74. The random forest model demonstrated the same overall accuracy (0.69), but with an improved recall rate of 0.69 for the positive group and a slightly higher AUC of 0.75. The confusion matrix (**Figure 1**) indicated that while the random forest model enhanced sensitivity, it did so at the expense of a modest reduction in specificity. Comparative analysis of the ROC curves (**Figure 2**) showed similar overall performance between the two models.

**Table 5:** Classification report for logistic regression model.

Class	Precision	Recall	F1-Score	Support
Negative	0.67	0.78	0.72	45
Positive	0.71	0.60	0.65	42
Accuracy			0.69	87
Macro average	0.69	0.69	0.69	87
Weighted average	0.69	0.69	0.69	87

Negative and Positive classes correspond to non-bacteremia and bacteremia groups, respectively. Precision reflects the model’s ability to avoid false positives, Recall (sensitivity) measures its capacity to identify true positives, and F1-Score balances these metrics.

**Table 6:** Classification report for random forest model.

Class	Precision	Recall	F1-Score	Support
Negative	0.72	0.76	0.74	45
Positive	0.72	0.69	0.71	42
Accuracy			0.69	87
Macro average	0.72	0.72	0.72	87
Weighted average	0.72	0.72	0.72	87

Macro average refers to the unweighted mean of performance metrics across all classes, treating each class equally regardless of its frequency. Weighted average accounts for the number of instances in each class (support) and is especially relevant in imbalanced datasets.

## Discussion

This study investigated the early predictive value of routine hematological and metabolic biomarkers for bacteremia and compared the classification performance of logistic regression and random forest models. Based on clinical data from 287 hospitalized patients, we identified several biomarkers significantly associated with bacteremia and demonstrated that both machine learning models achieved moderate predictive performance. These findings support the potential utility of routine laboratory indicators as tools for early bacteremia screening.

### Independent risk factors

Multivariate logistic regression analysis identified elevated platelet count, PCT, and triglycerides, as well as decreased cholesterol levels, as independent predictors of bacteremia. Increased platelet count may reflect the systemic inflammatory response and coagulation activation during acute infection. Previous studies have associated reactive thrombocytosis with poor outcomes in sepsis (Presume et al., 2022). With respect to procalcitonin (PCT), levels ≥ 0.5 ng/mL are generally regarded as indicative of systemic bacterial infection, while levels >2 ng/mL are associated with higher likelihood of sepsis (Jongwutiwes et al., 2009; Kubo et al., 2024). In our cohort, the median PCT level in bacteremia cases was 0.202 ng/mL (IQR: 0.094 - 0.771), suggesting that even modest elevations may carry predictive value when combined with other biomarkers. This underscores the importance of integrating multiple parameters rather than relying on a single threshold.

Our findings that low cholesterol and elevated triglycerides were independently associated with bacteremia are consistent with the phenomenon of infection-induced metabolic dysregulation (Górecka et al., 2022). Inflammatory cytokines such as interleukin-6 (IL-6) and tumor necrosis factor-alpha (TNF-α) are known to promote hypertriglyceridemia through enhanced hepatic lipogenesis and reduced lipoprotein lipase activity, while simultaneously suppressing cholesterol synthesis and increasing cholesterol catabolism (Górecka et al., 2022; Agnello et al., 2021). Similarly, stress-induced hyperglycemia observed in systemic infections reflects cytokine-driven insulin resistance and increased gluconeogenesis (Leonidou et al., 2008). These mechanisms highlight the pathophysiological basis for the observed metabolic alterations.

Although hyperglycemia did not reach statistical significance in the multivariate analysis (p = 0.067), its borderline association suggests that it should still be considered a clinically relevant risk factor. Bacteremia-induced insulin resistance and enhanced gluconeogenesis may lead to stress-induced hyperglycemia, which can exacerbate organ dysfunction and negatively impact prognosis (Leonidou et al., 2008). Similarly, decreased levels of hemoglobin and albumin, which were significant in univariate analysis, may reflect chronic inflammation, malnutrition, or bone marrow suppression commonly observed in patients with bacteremia (Allison and Lobo, 2024).

### Model performance and clinical applicability

The random forest model demonstrated superior sensitivity in detecting positive cases (recall = 0.69) compared to logistic regression (recall = 0.60), with a slightly higher AUC (0.75 vs. 0.74). This suggests that random forest may be more suitable in clinical settings prioritizing sensitivity, such as early screening. In contrast, logistic regression provides interpretable coefficients and stable performance, which may facilitate risk communication and clinical decision-making (Agnello et al., 2024). Our findings are consistent with previous reports. For example, prior machine learning studies, including those by Hernandez et al (Hernandez et al., 2025), incorporated both clinical and laboratory data and achieved AUCs up to 0.83 - 0.90, outperforming our models.

Several established tools, such as qSOFA, SIRS, and NEWS, also rely on physiological parameters that are not captured in our laboratory-based model. The primary advantage of our approach lies in its reliance on inexpensive, routine laboratory tests that can be automated and rapidly obtained, particularly useful in resource-limited settings where clinical scoring may be incomplete. However, the exclusion of physiological data limits our model’s discriminative power, highlighting the need for future multimodal model development.

### Future perspectives

Further research should aim to: Incorporate multicenter datasets to enhance model adaptability and robustness. Integrate multimodal data, including clinical scores, microbiological profiles, and imaging features, to improve predictive accuracy. Explore advanced algorithms and interpretability tools, such as XGBoost, LightGBM, SHAP, and LIME, to balance predictive performance with clinical transparency.

## Conclusion

In this study, we developed two machine learning models—logistic regression and random forest—to predict bacteremia based on routine hematological and metabolic indicators. Our findings identified elevated platelet count, procalcitonin, and triglycerides, along with decreased cholesterol levels, as independent risk factors for bacteremia. Both models achieved comparable performance with moderate predictive accuracy, while the random forest model demonstrated slightly better sensitivity for identifying positive cases.

The integration of machine learning algorithms with widely available laboratory parameters offers a cost-effective and accessible approach to support early detection of bacteremia, especially in clinical settings where rapid microbiological confirmation is limited. Nonetheless, to improve model generalizability and precision, future work should focus on incorporating high-dimensional clinical, physiological, and molecular data, and validating the models in multicenter prospective cohorts.

## Limitations

This study has several limitations. First, as a pilot single-center study with a modest sample size, the statistical power is limited, which may affect the stability and reliability of the predictive models. Second, the restricted time frame may limit the generalizability of the findings, as temporal variability in patient populations or clinical practices was not captured. Third, the single-center design further constrains external validity because patient characteristics and infection epidemiology may differ across institutions and regions.

To address these limitations, we are planning to extend the study period in future research to include a broader range of data over time, which will allow evaluation of temporal trends and enhance model robustness. In addition, we are preparing a multicenter, prospective validation study across hospitals in Fujian Province and beyond, aiming to assess the generalizability, calibration, and clinical utility of our models in diverse healthcare settings. Such external validation will be essential before clinical implementation.

## Data Availability

The original contributions presented in the study are included in the article/supplementary material. Further inquiries can be directed to the corresponding author.
